# 本科分析化学实验“正反案例式教学”的探讨与实践：固相微萃取-高效液相色谱测定环境样品中的6种含氮农药

**DOI:** 10.3724/SP.J.1123.2025.10005

**Published:** 2026-06-08

**Authors:** Jie ZHANG, Xinzhong ZHANG, Zhen ZHANG, Jingwei LIU, Xuemei WANG

**Affiliations:** 高原交汇区水资源安全与水环境保护教育部重点实验室，甘肃省生物电化学与环境分析重点实验室，西北师范大学化学化工学院，甘肃 兰州 730070; Key Laboratory of Water Security and Water Environment Protection in Plateau Intersection，Ministry of Education，Gansu Provincial Key Laboratory of Bioelectrochemistry and Environmental Analysis，College of Chemistry and Chemical Engineering，Northwest Normal University，Lanzhou 730070，China

**Keywords:** 样品前处理, 固相微萃取, 教学实践, 正反式案例教学, 含氮农药, sample pretreatment, solid-phase microextraction （SPME）, teaching practice, positive-negative case-based teaching, nitrogen-containing pesticides

## Abstract

分析化学中的分离与富集步骤不仅是分析流程的关键环节，更是决定分析方法准确性、可靠性及适用性的核心技术。然而，在传统实验教学中，该环节常因课时限制和教学重心偏重仪器操作而遭到忽视，导致学生知识体系呈现“碎片化”，难以建立理论联系实际的完整分析科学思维。为解决这一问题，本文立足“知识-能力-实践”三维融合的教学目标，以固相微萃取（SPME）技术萃取环境水样中6种含氮农药为案例，创新性构建“正反案例式教学”模式。通过对比非极性聚二甲基硅氧烷（PDMS）涂层与强极性聚丙烯酸酯（PA）涂层的纤维，采用高效液相色谱-紫外检测法（HPLC-UV），系统探究分析物极性与涂层性质的匹配规律：正向案例中PDMS涂层基于“相似相溶”原理，对弱极性农药展现出更优的萃取性能（线性决定系数*R*^2^≥0.993 7，检出限0.019~0.17 μg/L）；反向案例中PA涂层因萃取机理差异，萃取效果较弱（检出限0.066~1.069 μg/L）。结合对兰州市实际河流水样的加标回收试验（加标回收率为81.5%~117%），同步建立“知识内化-能力提升-实践素养”多维度评估体系。教学反馈显示，学生对核心原理的理解显著深化，创新思维与问题解决能力得到有效培养。“正反案例对比”教学能够有效衔接理论与实验、激发学生探究思维，为分析化学实验教学改革提供了可复制、可推广的实践范例，对培养具备理性方法选择与问题解决能力的高素质人才具有重要价值。

## Abstract (en)

In analytical chemistry， separation and enrichment steps serve not only as critical stages in the analytical workflow， but also as core technologies that determine the accuracy， reliability， and applicability of analytical methods. However， in conventional experimental instruction， this step is frequently overlooked owing to time constraints and an overemphasis on instrumental techniques， leading to fragmented knowledge structures and hindering the development of integrated analytical thinking that connects theory with practice. To address this issue， this study， based on the “knowledge-ability-practice” three-dimensional integrated teaching objective， innovatively establishes a “positive-negative case-based teaching” model， using the solid-phase microextraction （SPME） of six nitrogen-containing pesticides from environmental water samples as the instructional case. By comparing fibers coated with non-polar polydimethylsiloxane （PDMS） and strongly polar polyacrylate （PA）， and employing high performance liquid chromatography with ultraviolet detection （HPLC-UV）， this study systematically investigates the matching relationship between analyte polarity and coating properties. In the positive case， the PDMS coating demonstrates superior extraction performance for weakly polar pesticides based on the “like dissolves like” principle， with a linear coefficient of determination *R*^2^≥0.993 7 and detection limits ranging from 0.019 to 0.17 μg/L. In contrast， in the negative case， the PA coating shows weaker extraction efficiency due to differences in the extraction mechanism， yielding detection limits between 0.066 to 1.069 μg/L. Based on spiked recovery tests conducted on actual river water samples from Lanzhou （recoveries： 81.5% to 117%）， a multi-dimensional evaluation system of “knowledge internalization， competency enhancement， and practical literacy” is established accordingly. Teaching feedback demonstrates that the students show a marked improvement in their understanding of the core principles， and their innovative thinking and problem-solving skills are effectively cultivated. The “contrasting-case-based” pedagogical approach effectively bridges theory and experiment while stimulating students’ investigative thinking. The study provides a replicable and scalable practical model for the reform of analytical chemistry experimental teaching， which holds significant value in cultivating high-caliber talents with rational method selection and problem-solving abilities.

样品前处理作为分析过程中的关键环节^［1］^，旨在测定前对成分复杂的原始样品进行一系列处理，使其转化为适合仪器检测的形式。该环节通常包括采集、制备、分离、富集、净化及衍生化等步骤，以最大限度地减少基质干扰，提高目标分析物检测的灵敏度与准确性^［2，3］^。然而，传统的分析化学教学重点常偏重分析原理与仪器操作，导致学生对样品前处理的认识不足，难以应对实际科研与工业应用中复杂样品的分析挑战^［4-6］^。因此，如何改革教学内容与方法，充分发挥样品前处理环节的教学价值，已成为分析化学教育工作者亟待探讨的课题^［7］^。

案例教学法是由教师引导学生查阅文献、组织分组研讨、辅助总结经验的一类突出教学开放性与互动性的教学方法^［8］^。通过对典型案例的深度剖析，该教学方法可以促进学生知识内化与实践能力提升，有助于克服“理论空洞化”问题^［9］^。案例选取需兼顾典型性、启发性和前沿性，例如朱小红等^［10］^以“维生素C含量测定”氧化还原滴定实验为例，通过递进式问题设计，培养学生分析思维与解决问题的能力。

在环境分析领域，农药残留对水体安全构成威胁^［11］^，其痕量水平与基质复杂性使得样品前处理尤为关键^［12］^。固相微萃取（solid-phase microextraction， SPME）技术集采样、萃取与富集于一体，与色谱联用可实现高效、灵敏的检测，是理论与应用结合的良好教学载体^［13］^。

针对样品前处理教学中普遍存在的理论与实践脱节问题，本研究以SPME技术萃取环境水样中6种含氮农药为具体教学案例，在“知识-能力-实践”三维教学目标指引下，创新性地提出“正反案例式教学”模式。该模式将抽象理论转化为具体实验现象与数据，通过系统对比正向案例聚二甲基硅氧烷（PDMS）-SPME-高效液相色谱-紫外检测法（HPLC-UV）与反向案例聚丙烯酸酯（PA）-SPME-HPLC-UV的可行性与实用性，帮助学生直观理解方法选择的内在逻辑。同时，研究构建了覆盖知识内化、能力提升与实践素养的多维度教学评价体系，旨在强化学生理论应用能力，培养科研思维与创新意识，为行业需求导向的分析化学人才培养提供支持。

## 1 正反案例教学实验

### 1.1 实验教学目的及难点

实验教学目的：使学生掌握样品前处理的基本原理、分类、应用以及在分析化学中的重要性；通过实验探究，掌握SPME的结构、工作原理与涂层选择原则。本文设计“正反对比案例”，引导学生在分析化学实验过程中深化理解与灵活运用相关知识。

实验教学难点：在于引导学生突破表观现象，建立从宏观性能到微观机理的完整认知链条。在理论层面，要求学生结合聚合物化学结构及分子间作用力，阐释PDMS与PA涂层萃取性能差异的本质原因；在教学实践层面，基于PA涂层萃取效果不佳的实验结论，引导学生依据分离原理提出改进方案，实现从理论认识到方法创新的能力提升。

### 1.2 实验教学安排

实验前，学生需要通过线上平台观看SPME原理以及操作演示视频，预先了解如何规范使用高效液相色谱仪。实验以4~5人为一组，各组需明确分工，协作完成正反案例的对比探究。实验总学时数为8学时，具体包括：SPME基本原理与操作规范讲解（1学时）、PDMS涂层对6种农药的萃取与检测（正向案例，3学时）、PA涂层对同系列农药的萃取与检测（反向案例，3学时）、两组数据对比与机理研讨（1学时）。实验结束后，各小组应结合色谱数据与“相似相溶”原理，讨论PDMS与PA涂层极性与分析物性质的匹配规律，完成实验报告。在整个教学实施中，教师要在学生预测、操作、讨论的关键节点予以引导和反馈。

### 1.3 实验案例导入——正、反向案例教学

正向案例：PDMS属于非极性或弱极性聚合物，其硅氧键的极性因甲基基团的对称性与可旋转性被显著削弱，分子偶极矩较小，疏水性强^［14］^。本案例选取以PDMS为涂层的SPME商业纤维丝作为环境水体中6种农药的检测载体。

教学中，首先向学生阐明SPME的核心原理在于目标物在样品基质与萃取涂层间的“分配平衡”，并由此引出“如何利用该原理高效富集水环境中痕量农药”的关键问题。学生通过操作非极性PDMS涂层纤维萃取6种农药，直观验证其富集效果；随后，引导学生将实验数据与理论知识相衔接：通过分析农药分子中疏水性芳香环或长烷基链的结构特征，学生自主推论出“相似相溶”原则在此场景下的具体体现，即弱极性农药倾向于从极性水相分配至非极性PDMS涂层，从而将抽象的“分配平衡”原理转化为具体的认知。教学重点进一步升华至批判性思维与科学素养的培养，组织学生依据实验数据与操作体验，综合探讨PDMS对目标物传质效率、最终分配平衡以及纤维涂层稳定性的潜在影响，使学生实现从原理理解、技能掌握到方案优化能力的全面深化。

反向案例：PA分子中含有大量极性较强的酯基（-COO-）的侧链。酯基中的羰基（C=O）氧原子的电负性高，导致电子云强烈地偏向氧原子一侧，分子表现出较强的极性。此外，酯基中的氧原子可以作为氢键受体，与水或其他含有活泼氢的极性分子形成氢键，从而增强了其与目标物之间的分子间作用力。本案例中使用活化后的商业PA涂层SPME纤维丝作为环境水体中6种农药的检测装置，其余实验条件与正向案例一致。

在该案例中，需引导学生从分子间作用力的机理层面深入分析：PA涂层的强极性使其主要依靠氢键、偶极-偶极及*π-π*等特异性相互作用，而这与弱极性农药分子之间的作用力很弱；相比之下，PDMS涂层基于“相似相溶”原理，通过非极性的分配作用对疏水性化合物表现出普适而高效的萃取能力。由此，学生应认识到PA涂层的设计初衷是选择性捕获带有极性官能团的化合物（如酚类、羧酸类等），其在本实验中对弱极性农药的“低效”，源于分析物与涂层之间的机理不匹配。这最终帮助学生建立起深刻认识：涂层性能并非绝对，关键取决于其与分析物之间作用机制是否相适应。

### 1.4 仪器、试剂与材料

Agilent 1260高效液相色谱（美国安捷伦科技有限公司）；TP-214电子分析天平（丹佛仪器（北京）有限公司）；KQ3200E超声波清洗器（昆山市超声仪器有限公司）；ULTRAplus热场发射扫描电镜（德国Zeiss公司）；B11-2恒温磁力搅拌器（上海司乐仪器有限公司）；TG165离心机（长沙平凡仪器仪表有限公司）；PDMS、PA纤维（美国Supelco公司）。

甲醇（优级纯，赛默飞世尔科技（中国）有限公司）；氯化钠（分析纯，国药集团化学试剂有限公司）；盐酸（分析纯，国药集团化学试剂有限公司）；所用超纯水均由优普系列超纯水净化器（四川优普超纯科技有限公司）制备。

农药标准溶液的配制：以甲醇为溶剂，分别称取1.0 mg的6种农药（如表1）标准品，在容量瓶中定容成100.0 mg/L的标准储备液。

**表1:** 6种含氮农药的结构及相关性质

Classification	Chemical	Structure	CAS No.	*M*_r_	Polarity
Fungicide	chlorothalonil （百菌清）	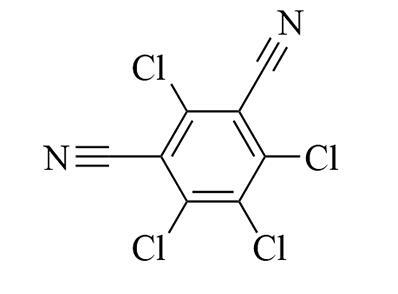	1897-45-6	265.9	intermediate
Fungicide	tebuconazole （戊唑醇）	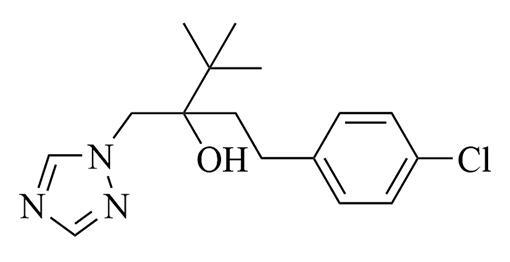	107534-96-3	307.8	intermediate
Caricide	chlorpyrifos （毒死蜱）	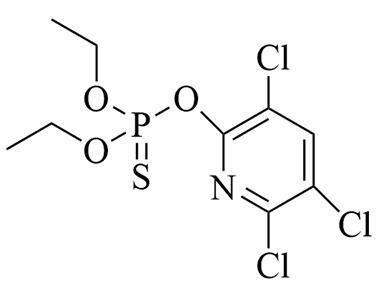	2921-88-2	350.6	weak
Herbicide	butralin （仲丁灵）	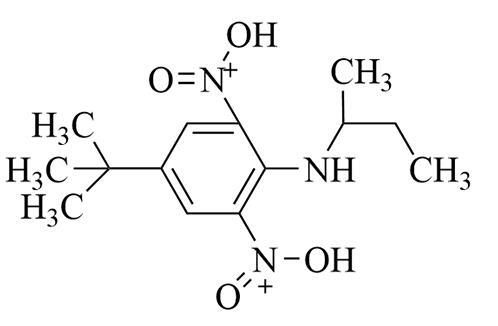	33629-47-9	295.3	weak
Insecticide	deltamethrin （溴氰菊酯）	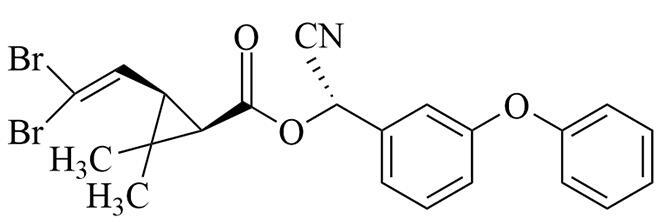	52918-63-5	505.2	weak
Acaricide	pyridaben （哒螨灵）	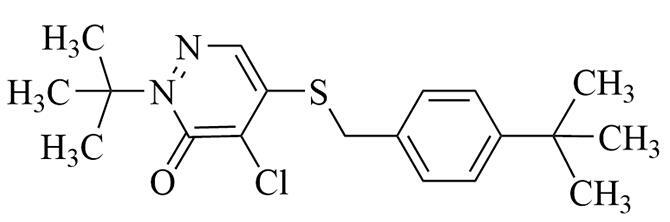	96489-71-3	364.9	weak

标准工作液的配制：将标准储备液精准移取到特定规格的容量瓶中，用超纯水稀释成所需不同浓度的标准工作液。

实际水样的制备：在甘肃省兰州市某河流上、中、下游分别采集3份水样，经0.45 µm有机膜过滤，去除不溶性杂质，重复操作3次。

以上溶液和实际水样置于4 ℃下避光保存。

### 1.5 萃取步骤及色谱条件

萃取步骤：将PDMS（或PA）纤维插入15.0 mL农药标准溶液中，在最优条件^［15］^下（萃取温度45 ℃、搅拌速率350 r/min、萃取时间25 min），完成萃取过程，随后将纤维置于含300.0 μL甲醇的棕色琥珀瓶中静态解吸10 min，将最后得到的洗脱液经滤膜过滤后进样分析。

色谱条件：SunFire C18色谱柱（150 mm×4.6 mm，5 μm，美国Waters公司）；流动相A为甲醇，B相为超纯水。紫外检测波长为282 nm，发射波长为389 nm，进样量为10 μL，柱温为25 ℃。

## 2 结果与讨论

### 2.1 PDMS纤维和PA纤维的结构表征

两种不同极性的纤维如图1所示，采用扫描电子显微镜（SEM）对PDMS与PA两种SPME纤维涂层的表面形貌进行表征，如图1a、1b所示。在SEM表征图中，两种纤维均呈现出光滑且均匀的形貌（图1c、1d）。

**图1:**
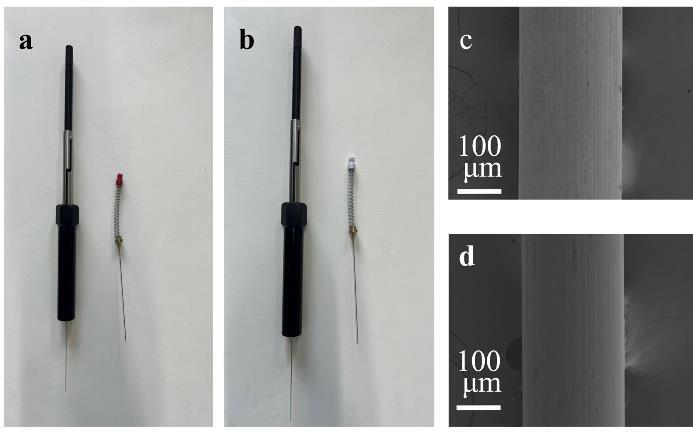
（a）PDMS纤维及SPME手柄，（b）PA纤维及SPME手柄，（c）PDMS纤维的SEM图，（d）PA纤维的SEM图

### 2.2 PDMS和PA纤维的性能分析和方法评估

为了进一步验证该方法的可行性，在最佳的SPME条件下，分别对正向案例（PDMS-SPME-HPLC-UV）和反向案例（PA-SPME-HPLC-UV）方法的线性范围、线性决定系数（*R*^2^）、检出限（LOD）和定量限（LOQ）等分析参数进行分析评价。如表2所示，正向案例所建立的方法在0.06~200 μg/L范围内，6种含氮农药的质量浓度与色谱峰面积呈现出良好的线性关系，*R*^2^值均大于0.993 7，LOD（信噪比（*S/N*）=3）和LOQ（*S/N=*10）分别为0.019~0.17 μg/L和0.057~0.510 μg/L。单根纤维方法的日内相对标准偏差（RSD）和日间RSD分别为1.63%~4.43%和4.07%~8.31%。此外，纤维-纤维重复性（RSD， *n*=5）为5.87%~7.75%。

**表2:** 所建立SPME-HPLC-UV方法的分析参数（*n=*5）

Analyte	Type of fiber	Linear range/ （μg/L）	*R*^2^	Precisions （RSDs/%）		Fiber-to-fiber reproducibility （RSD/%）	LOD/（μg/L）	LOQ/ （μg/L）
				Intra-day	Inter-day			
Chlorothalonil	PDMS	0.06-200	0.9991	3.45	4.27	5.87	0.019	0.057
	PA	0.30-200	0.9985	4.16	6.40	7.60	0.066	0.220
Tebuconazole	PDMS	0.20-200	0.9945	1.94	5.13	6.22	0.045	0.132
	PA	0.80-200	0.9979	4.76	7.44	6.70	0.229	0.763
Chlorpyrifos	PDMS	0.10-200	0.9937	3.14	8.31	7.70	0.032	0.096
	PA	0.70-200	0.9911	5.71	7.95	8.40	0.205	0.684
Butralin	PDMS	0.30-200	0.9986	2.51	4.67	7.54	0.075	0.225
	PA	0.40-200	0.9849	5.47	6.75	8.34	0.098	0.326
Deltamethrin	PDMS	0.60-200	0.9939	4.43	6.44	7.75	0.170	0.510
	PA	3.60-200	0.9931	4.32	5.27	9.10	1.069	3.565
Pyridaben	PDMS	0.20-200	0.9949	1.63	4.07	6.05	0.061	0.183
	PA	1.10-200	0.9985	2.60	5.70	6.70	0.315	1.052

反向案例所建立的方法在0.30~200 μg/L围内，*R*^2^均大于0.984 9，LOD（*S/N*）=3）和LOQ（*S/N*=10）分别为0.066~1.069 μg/L和0.220~3.565 μg/L。单根纤维方法的日内RSD和日间RSD分别为2.60%~5.71%和5.27%~7.95%。此外，纤维-纤维重复性（RSD，*n*=5）为6.70%~9.10%。

在该案例实验中，正向案例方法在灵敏度、线性及精密度方面均优于反向案例方法，显示出更可靠的分析性能，更适合对6种含氮农药进行痕量检测和定量分析。

### 2.3 实际水样分析

为了验证所建立方法的可行性，对实际采集水样中6种含氮农药进行定量分析，实验结果如表3所示，仅正向案例方法检测到河流上游中痕量的戊唑醇、毒死蜱、仲丁灵。为了评估所建立方法的准确度和精密度，在环境水样中分别加入10.0 μg/L和20.0 μg/L的6种含氮农药标准溶液。实验结果表明，正向案例中，所建立的方法对环境水样中6种含氮农药检测的加标回收率为81.5%~117%，RSD为1.06%~8.65%。反向案例中，加标回收率为82.2%~113%，RSD为1.92%~8.21%。表明所建立的两种方法均对6种含氮农药的分析具有较高的准确度和精密度，可用于环境水样中痕量含氮农药的预富集和检测。

**表3:** 环境水样中的6种含氮农药的分析结果（*n*=3）

Sample	Analyte	Type of fiber	Original/（μg/L）	Spiked with 10.0 μg/L				Spiked with 20.0 μg/L		
				Detected/（μg/L）	Recovery/%	RSD/%		Detected/（μg/L）	Recovery/%	RSD/%
The upper reaches of river	chlorothalonil	PDMS	ND	10.8	81.5	5.65		20.5	102	5.69
		PA	ND	8.96	89.7	3.98		17.5	87.5	5.51
	tebuconazole	PDMS	2.66	10.7	81.5	7.79		19.8	99.1	5.11
		PA	ND	11.3	113	3.64		27.2	82.2	3.37
	chlorpyrifos	PDMS	3.43	14.4	109	2.63		23.7	101	1.67
		PA	ND	9.81	98.1	5.62		21.6	108	4.45
	butralin	PDMS	3.51	13.1	96.7	2.19		23.9	102	1.09
		PA	ND	9.81	98.1	5.62		21.6	108	4.45
	deltamethrin	PDMS	ND	9.34	93.4	8.65		20.4	87.1	6.54
		PA	ND	8.74	87.4	7.98		19.2	96.2	8.21
	pyridaben	PDMS	ND	11.0	110	2.52		19.5	97.3	2.80
		PA	ND	9.70	97.0	5.43		22.2	111	6.45
The middle reaches of river	chlorothalonil	PDMS	ND	10.2	102	1.94		23.9	109	2.35
		PA	ND	8.39	83.9	4.98		18.6	93.0	5.72
	tebuconazole	PDMS	ND	8.15	81.5	4.92		21.8	108	2.24
		PA	ND	9.98	99.8	2.34		18.2	90.8	3.79
	chlorpyrifos	PDMS	ND	10.3	101	8.06		20.3	102	5.13
		PA	ND	10.4	104	5.76		20.1	101	3.74
	butralin	PDMS	ND	11.7	117	5.84		22.4	112	6.43
		PA	ND	8.94	89.5	3.74		20.2	101	3.89
	deltamethrin	PDMS	ND	10.5	105	6.63		21.4	107	8.02
		PA	ND	9.34	93.4	4.76		19.4	97.0	2.32
	pyridaben	PDMS	ND	10.7	107	4.46		21.7	108	6.93
		PA	ND	9.16	91.6	5.69		21.3	106	1.92
The lower reaches of river	chlorothalonil	PDMS	ND	9.15	91.5	4.50		20.8	104	2.48
		PA	ND	9.78	97.5	6.61		17.8	89.1	5.05
	tebuconazole	PDMS	ND	11.5	115	4.35		20.3	101	2.99
		PA	ND	8.58	85.8	7.98		19.3	96.4	2.49
	chlorpyrifos	PDMS	ND	10.9	109	6.01		19.5	93.5	3.58
		PA	ND	10.0	100	2.55		18.3	91.4	2.19
	butralin	PDMS	ND	10.4	104	5.27		19.3	96.5	4.83
		PA	ND	8.98	89.8	3.65		19.8	98.9	2.65
	deltamethrin	PDMS	ND	9.23	92.3	5.45		19.5	97.7	2.91
		PA	ND	10.3	103	6.83		18.8	94.1	3.46
	pyridaben	PDMS	ND	11.5	115	3.90		20.4	102	3.71
		PA	ND	9.86	98.6	7.11		19.1	95.8	7.52

ND： not detected or lower than LODs.

在完成定量分析的基础上，进一步引导学生对实验结果进行深度解析。通过对比分析加标水平为20.0 μg/L的含氮农药标准溶液在实际水样中的色谱图（图2），可以直观地发现PA涂层纤维的色谱峰高远低于PDMS涂层纤维。这表明PA涂层纤维对多种目标农药的萃取能力显著弱于PDMS涂层。以此为切入点，我们通过这一正一反的典型案例，引导学生理解两者在萃取机理上的根本差异。PA纤维头的萃取过程更倾向于一种竞争性的吸附机理^［16］^，其有限的极性作用位点会受水样中共存极性物质的干扰；而PDMS纤维头则主要遵循非竞争性的分配/吸收机理，其对弱极性有机物的溶解作用具有更广泛的适用性和抗基质干扰能力。通过上述正反案例的对比分析，学生得以从宏观现象深入到微观作用机理与热力学过程，最终牢固建立起“依据分析物极性理性选择适配涂层”的科学思维。

**图2:**
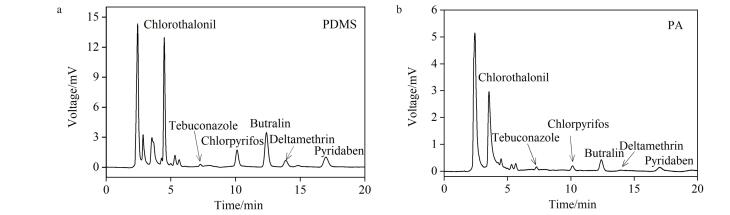
（a）PDMS纤维和（b）PA纤维的SPME-HPLC图

## 3 教学反馈及教学效果的多维度评估

### 3.1 教学反馈

为客观评估“正反案例式教学”模式的效果，本研究通过问卷调查、学生访谈及报告分析等多种形式收集并分析了教学反馈。评估结果表明，该模式显著增强了学生对课程内容的理解深度，有效促进了其对核心知识的整合与掌握，整体教学效果达到预期目标。

### 3.2 教学效果的多维度评估

打破“以实验报告为主”的单一评估模式，建立“知识-能力-实践”三维评估体系^［17，18］^，全面检验教学效果。

首先，在知识评估方面，我们着重考查学生对核心原理的内化与迁移能力。具体通过在完成SPME正反案例学习后，布置原理应用题（如“阐述PDMS与PA涂层萃取机理的根本差异，并解释其对6种农药回收率的影响”）和方案设计题（如“针对一类新型极性农药，基于‘相似相溶’原理设计前处理方案并阐明理由”），从而评估学生对聚合物结构、极性、分子间作用力等理论知识的掌握深度与应用灵活性。

其次，在能力评估方面，我们通过实验操作考核，从“操作规范性（如SPME纤维活化、萃取、解吸流程）、结果准确性（如回收率）、问题解决能力（如处理实验异常）”3个维度进行评分。实验报告采用“方法优化建议”和“新型技术探索报告”等方法，综合评估学生的创新意识与能力。

最后，在实践评估方面，我们注重学生严谨科学态度与规范意识的培养。通过检查实验记录的规范性和评估废液处理的安全意识，于细节中夯实其科研基本功。同时，在教学中有机融入行业标准并进行相关考核，引导学生建立分析检测规范流程的意识，实现从单纯完成实验到理解行业要求的升华。

## 4 结论

本研究针对本科分析化学实验中样品前处理教学被忽视的问题，以SPME检测环境水样中6种含氮农药为载体，创新构建“正反案例式教学”模式——通过对比非极性PDMS涂层与强极性PA涂层的SPME-HPLC-UV检测效果，结合实际水样验证，明确分析物极性与涂层性质的匹配规律及萃取机理差异；同时建立“知识-能力-实践”三维评估体系，教学反馈显示学生深化了核心原理的理解。该模式破解了传统教学理论与实践脱节问题，实现了知识传递、能力培养与素养提升的协同，为分析化学实验教学改革提供了可复制案例，助力契合行业需求的人才培养。
